# Integrating network pharmacology and experimental verification to reveal the anti-inflammatory ingredients and molecular mechanism of pycnogenol

**DOI:** 10.3389/fphar.2024.1408304

**Published:** 2024-06-26

**Authors:** Hongyu Liu, Jie Shi, Fei Liu, Litao Zhang

**Affiliations:** School of Biological Science, Jining Medical University, Rizhao, Shandong, China

**Keywords:** anti-inflammation, network pharmacology, pycnogenol, UPLC-MS/MS, molecular mechainsm

## Abstract

**Introduction:** Pycnogenol (PYC), a standardized extract from French maritime pine, has traditionally been used to treat inflammation. However, its primary active components and their mechanisms of action have not yet been determined.

**Methods:** This study employed UPLC-MS/MS (Ultra-high performance liquid chromatography-tandem mass spectrometry) and network pharmacology to identify the potential active components of PYC and elucidate their anti-inflammatory mechanisms by cell experiments.

**Results:** 768 PYC compounds were identified and 19 anti-inflammatory compounds were screened with 85 target proteins directly involved in the inflammation. PPI (protein-protein interaction) analysis identified IL6, TNF, MMP9, IL1B, AKT1, IFNG, CXCL8, NFKB1, CCL2, IL10, and PTGS2 as core targets. KEGG (Kyoto Encyclopedia of Genes and Genomes) enrichment analysis suggested that the compound in PYC might exert anti-inflammatory effects through the IL17 and TNF signal pathways. Cell experiments determined that PYC treatment can reduce the expression of IL6 and IL1β to relieve inflammation in LPS (lipopolysaccharide)-induced BV2 cells.

**Conclusion:** PYC could affect inflammation via multi-components, -targets, and -mechanisms.

## 1 Introduction

Inflammation is a critical physiological and pathological process that prevents diseases, removes necrotic and damaged tissues, and targets infectious pathogens (Rafiyan and Sadeghmousavi, 2023; D'Arcy, 2019). Normally, inflammation resolves quickly and benefits the host; however, when acute inflammation becomes chronic, it can lead to a harmful cycle of aberrant inflammatory responses and prolonged tissue damage, contributing to the development of conditions such as diabetes, neurological disorders, heart disease, and cancer (Alderton and Scanlon, 2021; Langworth-Green et al., 2023; Nigam et al., 2023). Therefore, it is crucial to control inflammation through effective therapeutic strategies.

Pycnogenol (PYC) is a standardized extract from French maritime pine (*Pinus maritime*) comprising 65%–75% procyanidins. These procyanidins consist of varying chain-length subunits of epicatechin and catechin, along with other constituents such as flavonoids, polyphenolic monomers, and cinnamic or phenolic acids and their glycosides (Rohdewald, 2002). PYC exhibits immuno-modulatory, anti-inflammatory, antioxidant, and anti-carcinogenic effects (Maimoona et al., 2011). Furthermore, PYC has been traditionally used to treat various inflammatory diseases, including chronic, circulatory, and neurological conditions (Norouzy and Malekahmadi, 2023). Many *in vitro* studies have elucidated PYC’s anti-inflammatory mechanisms, including inhibition of proinflammatory cytokines and matrix metalloproteases, reduction of histamine release from mast cells, and blocking of inflammatory transcription factors (Cordova et al., 2013; Nattagh-Eshtivani et al., 2022).

Existing literature suggests that PYC significantly enhances antioxidant mediators, indicating that procyanidins may be its primary anti-inflammatory component (Shin et al., 2013). However, PYC also contains 25%–35% of other compounds, and the association of these ingredients with PYC’s anti-inflammatory effects remains unclear. Moreover, identifying and understanding the mechanisms of PYC’s anti-inflammatory compounds pose significant challenges. UPLC-MS/MS (Ultra-high performance liquid chromatography-tandem mass spectrometry) has high sensitivity, resolution, and selectivity; therefore, it has been utilized to characterize unknown trace components in herbal medicines rapidly (Wang et al., 2021). Network pharmacology, a novel interdisciplinary tool, merges aspects of pharmacology, computational and systems biology, network analysis, and bioinformatics to explore the relationships between traditional herbs and various diseases, facilitating the identification of signaling pathways and targets to enhance drug discovery efficiency holistically (Xian X et al., 2023). Therefore, integrating UHPLC-MS and network pharmacology offers a robust approach to assessing the anti-inflammatory components of PYC and their mechanisms.

**FIGURE 1:**
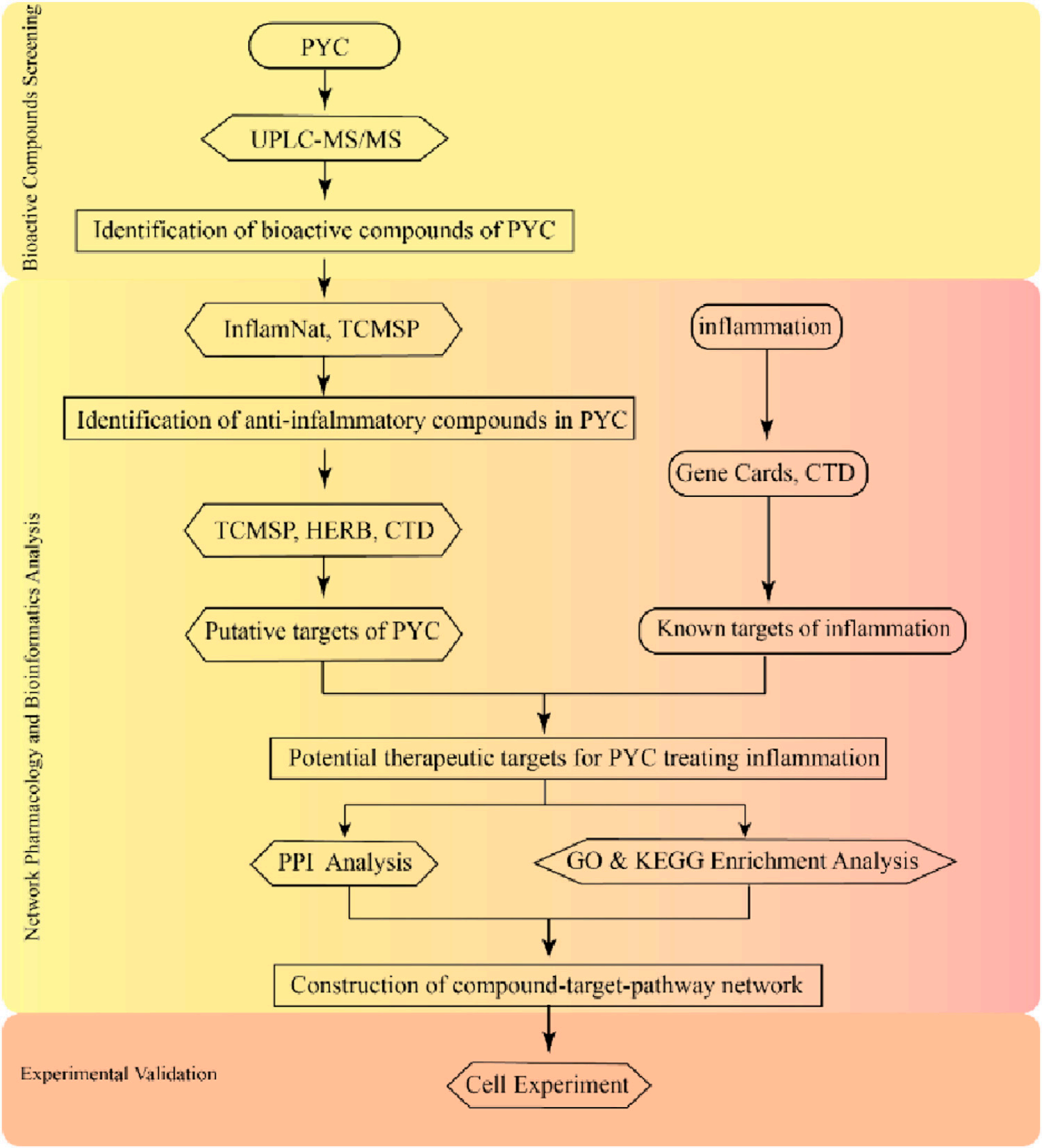
The flowchart of the experimental procedures in this study.

This investigation employed the UPLC-MS/MS to assess the active PYC compounds. Then, network pharmacology was used to identify key active targets, components, and potential signaling pathways related to the anti-inflammatory effects, enabling a detailed analysis of PYC’s anti-inflammatory mechanism. This research aims to provide evidence supporting the comprehensive evaluation and clinical application of PYC for treating inflammation. The experimental procedures of this study are depicted in Figure 1.

## 2 Material and methods

### 2.1 Materials

PYC was acquired from the Horphag Research Ltd. (Geneva, Switzerland). UNIQ-10 column total RNA purification kit and CCK8 (Cell Counting Kit-8) were bought from Sangon Biotech Co., Ltd. (Shanghai, China). MagicSYBR Mixture and HiFiScript cDNA Synthesis Kit were sourced from Jiangsu Cowin Biotech Co., Ltd (Nanjing, China). The BV 2 cell line and its specified media were brought from Procell Life Science &Technology Co., Ltd. (Wuhan, China). Lipopolysaccharides (LPS), Micro NO (nitric oxide) content and Malondialdehyde (MDA) content kits were obtained from Solarbio Science and Technology Co., Ltd. (Beijing, China).

### 2.2 Sample preparation

The PYC powder (2 g) was macerated with 50 mL double distilled H_2_O (dd H_2_O) in a 250 mL sample bottle and stored at 4°C for 12 h. This mixture was sonicated in an ice-cold water bath for 1 h and centrifuged at 4°C for 10 min at 10,000 × g to separate the buoyant fraction as sample (A), which was subsequently stored at 4°C. The remaining PYC residue was mixed with 30 mL of dd H_2_O, then sonicated, centrifuged, and collected as sample (B) using the abovementioned parameters. The extracted supernatant was then mixed and diluted to 100 mL with dd H_2_O as the final PYC sample for subsequent chemical analyses.

### 2.3 UPLC-MS/MS analysis

For chemical analysis, the UHPLC system (Vanquish, Thermo Fisher Scientific) fitted with a UPLC HSS T3 column (2.1 mm × 100 mm, 1.8 μm) coupled to an Orbitrap Exploris 120 mass spectrometer (Orbitrap MS, Thermo) was employed. The mobile phases included 5 mM/L acetic acid in acetonitrile and 5 mM/L ammonium acetate in water. The analysis was conducted at 40°C with a 0.4 mL/min flow rate.

With the help of the mass spectrometer, MS/MS spectra were obtained using the information-dependent acquisition (IDA) mode. The ESI source parameters were: 50 Arb flow rate of sheath gas, full MS resolution was 60000, 15 Arb flow rate of Aux gas, MS/MS resolution was 15000 collision energy as 10/30/60 in NCE mode, 320°C capillary temperature, spray Voltage was −3.4 kV (negative) or 3.8 kV (positive).

For 30 s, the extract supernatant (0.3 mL) and methanol extract solution (1 mL) were vortexed, then for 10 min was sonicated on ice-cold temperature before incubation for 1 h at −40°C and centrifugation at −40°C for 15 min at 12,000 rpm. The acquired supernatant was utilized for product assessment. The raw data extraction and chemical identification were based on the secondary and primary spectral data interpreted against the MS2 database. The UPLC-MS/MS analysis were commissioned by a professional biological company (Biotree DB, Shanghai).

### 2.4 Network pharmacology

The chemical components of PYC were characterized using UHPLC-MS. The compounds with anti-inflammatory bioactivity in PYC were identified using the Inflammatory Natural Products Database and Predictor (http://www.inflamnat.com/#/main/home). Compounds were further filtered based on ADME (absorption, distribution, metabolism, and excretion) properties from the Traditional Chinese Medicine Systems Pharmacology Database and Analysis Platform (TCMSP, https://tcmsp-e.com/index.php) with the criteria: drug-likeness (DL) ≥ 0.18, oral bioavailability (OB) ≥ 25%, and rotatable bond number (RBN) ≤ 10 (Li et al., 2022). Target predictions for these anti-inflammatory compounds were obtained from the TCMSP, High-throughput Experiment- and Reference-guided database of TCM (HERB, http://herb.ac.cn), and the Comparative Toxicogenomics Database (CTD, http://ctdbase.org). The human genes associated with the inflammation were screened from the Genecards database (https://www.genecards.org/) using the keyword “inflammation,” and to ensure results credibility, the relevance score was set to ≥5. The potential targets of the final anti-inflammatory compounds were assessed and compared with disease targets to acquire potential target genes for treating inflammation. Using the CTD, the common overlapping genes were depicted through the Venn diagram. Furthermore, these genes were uploaded to the STRING database (https://www.string-db.org/) to establish a protein-protein interaction (PPI) network and were graphically visualized using the Cytoscape (version 3.9.1). Moreover, Gene Ontology (GO) and Kyoto Encyclopedia of Genes and Genomes (KEGG) pathway enrichment assessments were conducted using the Database for Annotation, Visualization, and Integrated Discovery (DAVID, https://david.ncifcrf.gov/). The target organism was *Homo sapiens*, the false discovery rate (FDR)-adjusted *p-value* was employed for elucidating the enrichment analysis, and a *p-value* < 0.05 was termed significant.

### 2.5 Cell culture

Under the influence of various inflammatory factors, the BV2 microglial cell line can be over-activation and subsequently release inflammatory mediators such as TNFα, IL1β, and IL6, so here the BV2 microglial cell line were chosen for further study and was cultured at 37 °C in the BV2 cell specified media containing DMEM with 10% FBS (Fetal Bovine Serum) and 1% Penicillin-Streptomycin Solution, which is also the culture media for BV2 cells. Meanwhile, Both LPS and PYC were dissolved in BV2 cell specific media to obtain stocking solutions with concentrations of 10 μg/mL and 200 μg/mL, respectively.

### 2.6 Cell viability

BV2 microglial cells were seeded at 5,000 cells per well in a 96-well plate and cultured overnight in 100 μL of BV2 special medium. Then, for 24 h, the cells were treated with the PYC (at 100, 80, 60, 40, 20, 10, and 5 μg/mL concentrations) with and without LPS (1 μg/mL). The model group only received LPS (1 μg/mL) treatment, and the blank group received no treatment. For viability analysis, 10 μL of CCK-8 solution containing WST-8 (2-(2- methoxy-4-nitrophenyl)-3-(4-nitrophenyl)-5-(2,4-disulfophenyl)-2H-tetrazolium) was added to each well, and cells were incubated for 1 h at 37°C. The optical density (OD) was assessed using the Spark Multimode Reader Platform (Tecan, Switzerland) at 450 nm. The cell survival rate was elucidated as follows: Cell viability (%) = [(OD Sample-OD Blank)/(OD Control- OD Blank)] × 100%.

### 2.7 Cells treatments

BV2 cells were cultured at 37°C in a 6-well plate at a density of 2 × 10^5^ per well overnight in 5% CO_2_ using. Those cells were categorized into three groups: the blank (Control; received no treatment, the LPS (received BV2 special medium augmented with 1 μg/mL LPS), and the treatment (received BV2 special medium augmented with 80 μg/mL of PYC). The treatment time was 24 h. Each group contained four replicates.

### 2.8 Determination of cell physiological and biochemical indexes

The NO and MDA levels were quantified using commercial assay kits from Solarbio. The concentration of NO was determined using the Griess reagent. In short, 100 μL of cell lysis supernatant was combined with an equal volume of Griess reagent, containing a final concentration of 0.1% N-[1-naphthyl]ethylenediamine dihydrochloride in distilled water and 1% sulfanilamide in 5% phosphoric acid, in a 96-well flat-bottom plate. The absorbance was then measured at 550 nm after a 10-min incubation period. Finally, the content of NO was calculated from a standard curve plotted using sodium nitrite. The MDA concentration was assessed by TBA (Thiobarbituric Acid) method, and the reaction mixture included 100 μL of a cell lysis supernatant, 300 μL of MDA test fluid (containing TBA) and 100 μL of acetic acid. The mixed solution was incubated in a water bath at 100°C for 60 min, then tightly covered to prevent moisture loss. It was then cooled in an ice bath, centrifuged at 10,000 g for 10 min at room temperature. 200μL of supernatant was transferred to 96-well plate, and the absorbance of each sample at 532 nm was measured and the content of MDA can be estimated.

### 2.9 Measurement of proinflammatory cytokine production

The total cellular RNA was obtained with the help of the UNIQ-10 column total RNA purification kit, per the manufacturer’s guide. cDNA was prepared using the HiFiScript cDNA synthesis kit, followed by qPCR assessment *via* a CFX Connect Real-Time PCR Detection System (Bio-Rad, United States) using using a total 20 μL reaction volume containing 10 μL of Magic SYBR Mixture (2×), 4 μL of each primer (2 μM), and 2 μL of cDNA. The -ΔΔCq formula was applied to identify the target genes (IL6 and IL1β) expression. β-actin was set as an endogenous control. All qPCR primers are as follows, β-actin (Sense: GGC​TGT​ATT​CCC​CTC​CAT​CG, Antisense: CCA​GTT​GGT​AAC​AAT​GCC​ATG​T), IL1β (Sense: GCA​ACT​GTT​CCT​GAA​CTC​AAC​T, Antisense: ATC​TTT​TGG​GGT​CCG​TCA​ACT), IL6 (Sense: CTG​CAA​GAG​ACT​TCC​ATC​CAG, Antisense: AGT​GGT​ATA​GAC​AGG​TCT​GTT​GG).

### 2.10 Analysis of proinflammatory cytokine production

All assays were performed in quadruplicate, and the data are presented as mean ± standard error of the mean (SEM). The data assessment and statistical measurements were performed using the GraphPad Prism (9.0 version) software. The inter-group statistically significant variations were assessed using the one-way ANOVA followed by Duncan’s multiple range test. The significance threshold was set as *p* < 0.05.

## 3 Results

### 3.1 Screening of anti-inflammatory compounds in PYC

**FIGURE 2:**
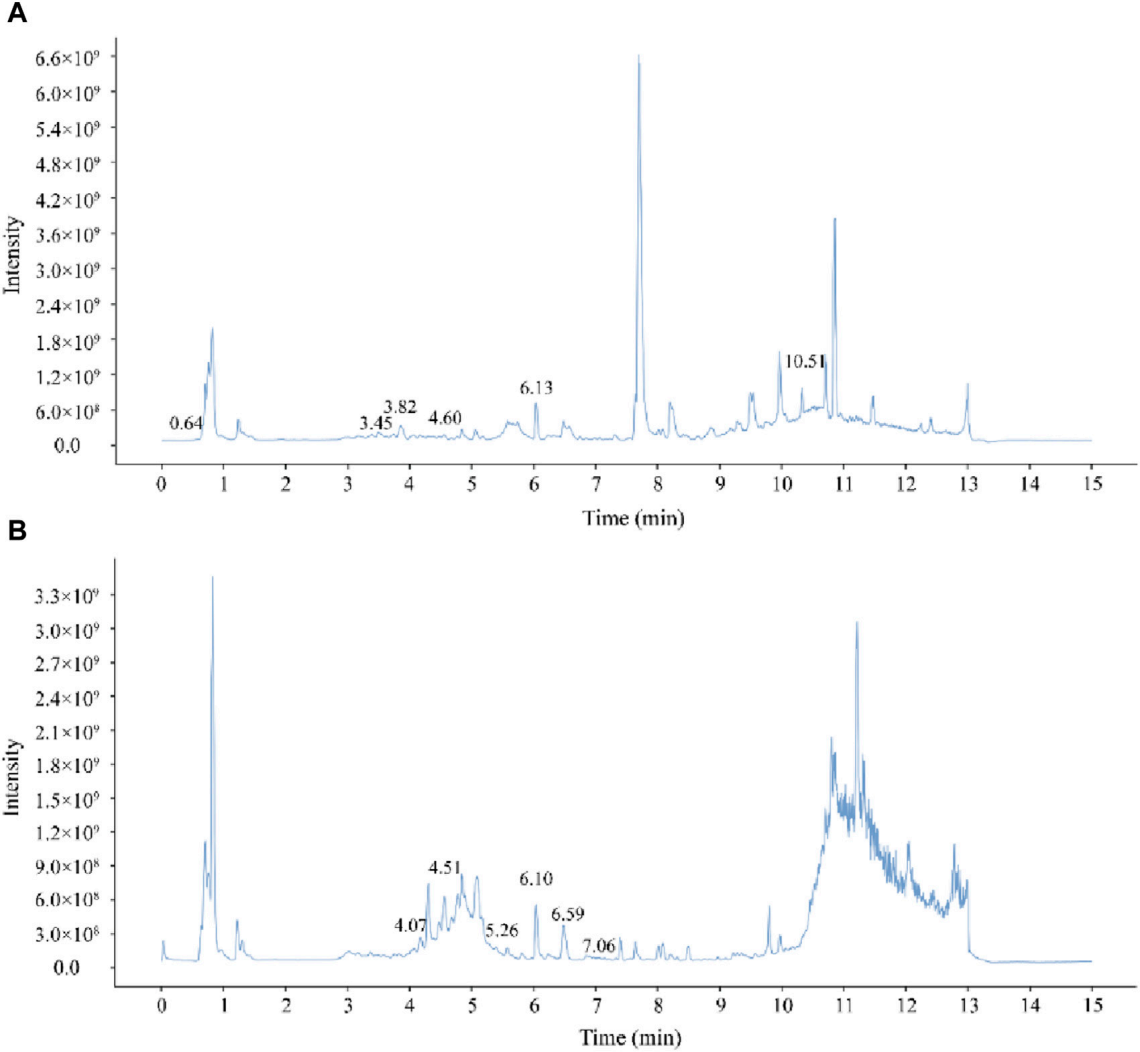
Mass spectrum chromatograms of PYC **(A)** Positive mode **(B)** Negative mode.

**FIGURE 3:**
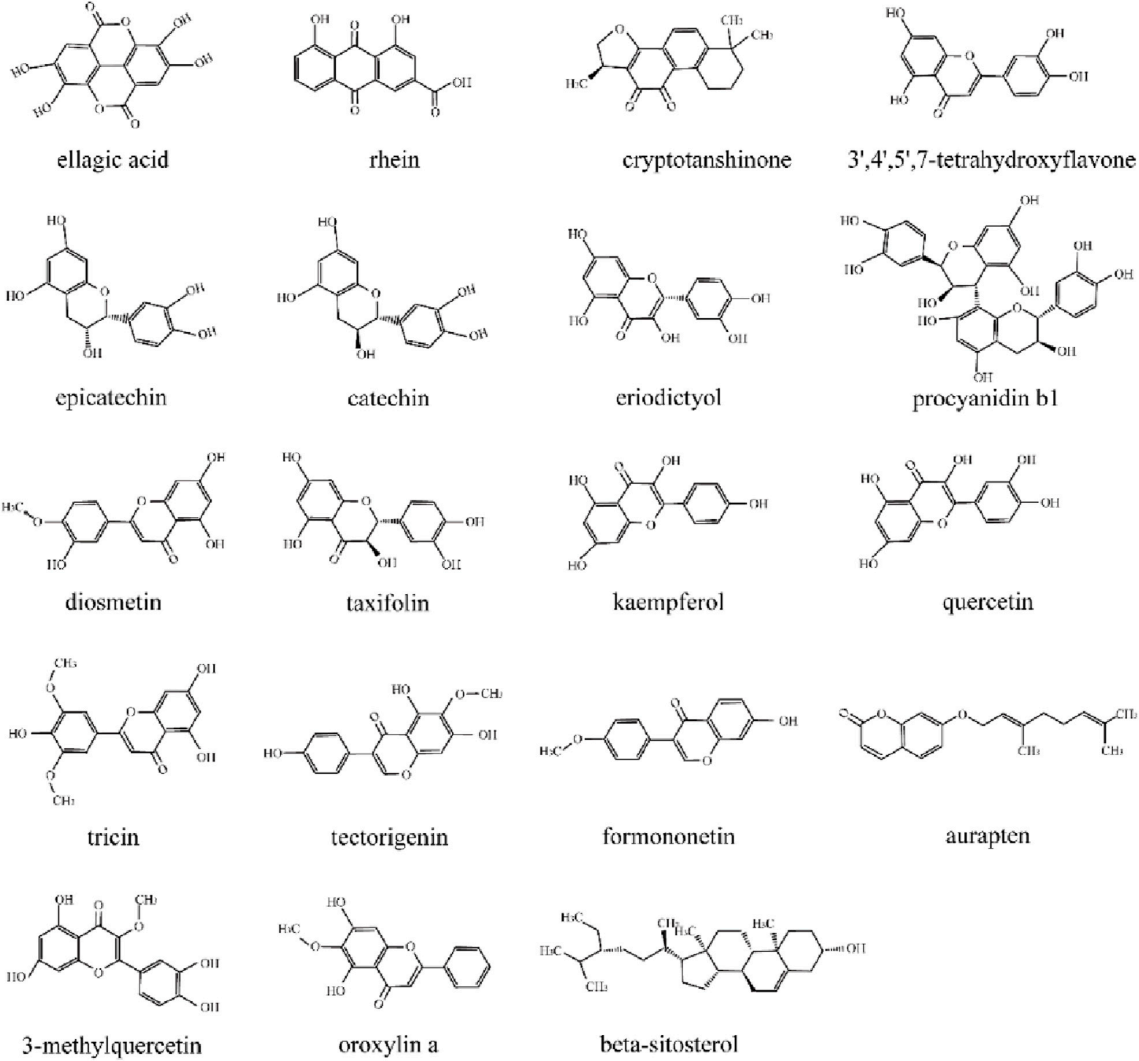
Chemical structure of anti-inflammatory compounds in PYC.

The chemical components of PYC were characterized using UHPLC-MS/MS. The mixed quality control sample’s total ions current was the sum of all ion’s intensities in the mass spectrum at each time point *versus* time (Figure 2). A total of 768 compounds were analyzed (Supplementary Table SA1). The InflamNat database indicated >1,351 (up to 2021) natural compounds with anti-inflammatory activity (Zhang et al., 2022). Using the InflamNat database, 53 chemical components in PYC were identified with anti-inflammatory bioactivity. Components were screened using the TCMSP database based on the following criteria: DL ≥ 0.18, RBN ≤10, and OB ≥ 25%. After eliminating repetitions, 19 anti-inflammatory compounds in PYC were screened (Table 1; Figure 3).

**TABLE 1:** Chemical information of anti-inflammatory compounds in PYC.

NO.	Retention time (s)	Name of compound	Formula	Molecular weight	Measured (m/z)	Adduct	ppm	Fragments (m/z)	OB (%)	DL	RBN	Relative abundance^[a]^
1	38.647	ellagic acid	C14H6O8	302	300.998734	[M-H]-	0.883872	300.999053 130.087893 300.193394 229.014242 283.995997	43.06	0.43	0	382579.041
2	65.5674	rhein	C15H8O6	284	283.028942	[M-H]-	0.987645	239.041526 266.146381 257.838318 256.033172	47.07	0.28	1	4,354.925
3	206.728	cryptotanshinone	C19H20O3	296	297.148201	[M+H]+	0.678162	297.145945 279.138606 299.059468 225.089625 251.143015	52.34	0.4	0	10386705.988
4	229.382	3′,4′,5,7-tetrahydroxyflavone	C15H10O6	286	287.054446	[M-H]-	0.572307	268.025699 251.08631 269.033766 252.023443 287.029103	36.16	0.25	1	77937.514
5	244.229	3-methylquercetin	C16H12O7	316	315.048998	[M-H]-	0.453271	283.040939 315.034873 298.024453 272.029305 270.484784	30.85	0.3	2	40997.763
6	270.58	eriodictyol	C15H12O6	288	289.070478	[M+H]+	1.655121	153.018803 289.070206 163.038279 171.028328 92.667316	71.79	0.24	1	1625231.223
7	272.274	procyanidin b1	C30H26O12	579	577.131042	[M-H]-	1.752171	560.107374 562.05499 494.06971 577.054087	67.87	0.66	3	16354099.728
8	273.009	epicatechin	C15H14O6	290	289.071264	[M-H]-	2.545758	178.99858 109.029346 151.003656 289.07314 125.024729	28.93	0.24	1	4407085.684
9	276.12	(+)catechin	C15H14O6	290	291.085946	[M+H]+	0.185453	139.038285 123.044269 165.054707 147.043595 179.072095	54.83	0.24	1	281573.580
10	277.932	diosmetin	C16H12O6	300	301.070460	[M+H]+	0.805349	282.364998 270.069685 301.085206 284.859801 256.814808	31.14	0.27	2	36056.858
11	315.576	taxifolin	C15H12O7	304	303.050743	[M-H]-	0.848278	183.028444 139.040525 165.019339 137.024378 97.02918	57.84	0.27	1	2292709.790
12	323.957	kaempferol	C15H10O6	286	285.040314	[M-H]-	1.102180	285.038328 257.045764 223.207939 267.197495 241.051217	41.88	0.24	1	218213.248
13	366.012	quercetin	C15H10O7	302	303.049586	[M+H]+	1.367047	303.047634 86.096161 257.043075 229.048736 165.018527	46.43	0.28	1	17541025.848
14	367.5155	oroxylin-a	C16H12O5	284	285.075492	[M+H]+	0.772107	268.036019 285.059018 211.041433 240.042973 269.040808	41.37	0.23	2	3661976.856
15	395.351	tricin	C17H14O7	330	329.066833	[M-H]-	0.508756	329.068561 299.018318 314.041901 271.022398 92.684248	27.86	0.34	3	936843.397
16	400.688	tectorigenin	C16H12O6	300	299.055776	[M-H]-	0.749747	299.057212 284.032462 240.042916 92.685104 149.995282	28.41	0.27	2	2134450.992
17	423.78	formononetin	C16H12O4	268	267.066305	[M-H]-	1.140565	267.064703 252.042868 223.040786 92.687614 268.069083	69.67	0.21	2	10699480.294
18	471.945	aurapten	C19H22O3	298	299.163634	[M+H]+	1.224780	163.038025 81.069768 95.085284 137.131947 119.048405	25.62	0.24	6	2710668.264
19	631.041	beta-sitosterol	C29H50O	415	397.379132	[M-H2O+H]+	0.620868	411.009614 410.956461 397.369983 91.0540917 70.0649644	36.91	0.75	6	5457708.356

[a] Chromatographic peak area presents the relative abundance of the anti-inflammatory compounds in PYC.

### 3.2 Targets prediction and establishment of compound-target-disease network

**FIGURE 4:**
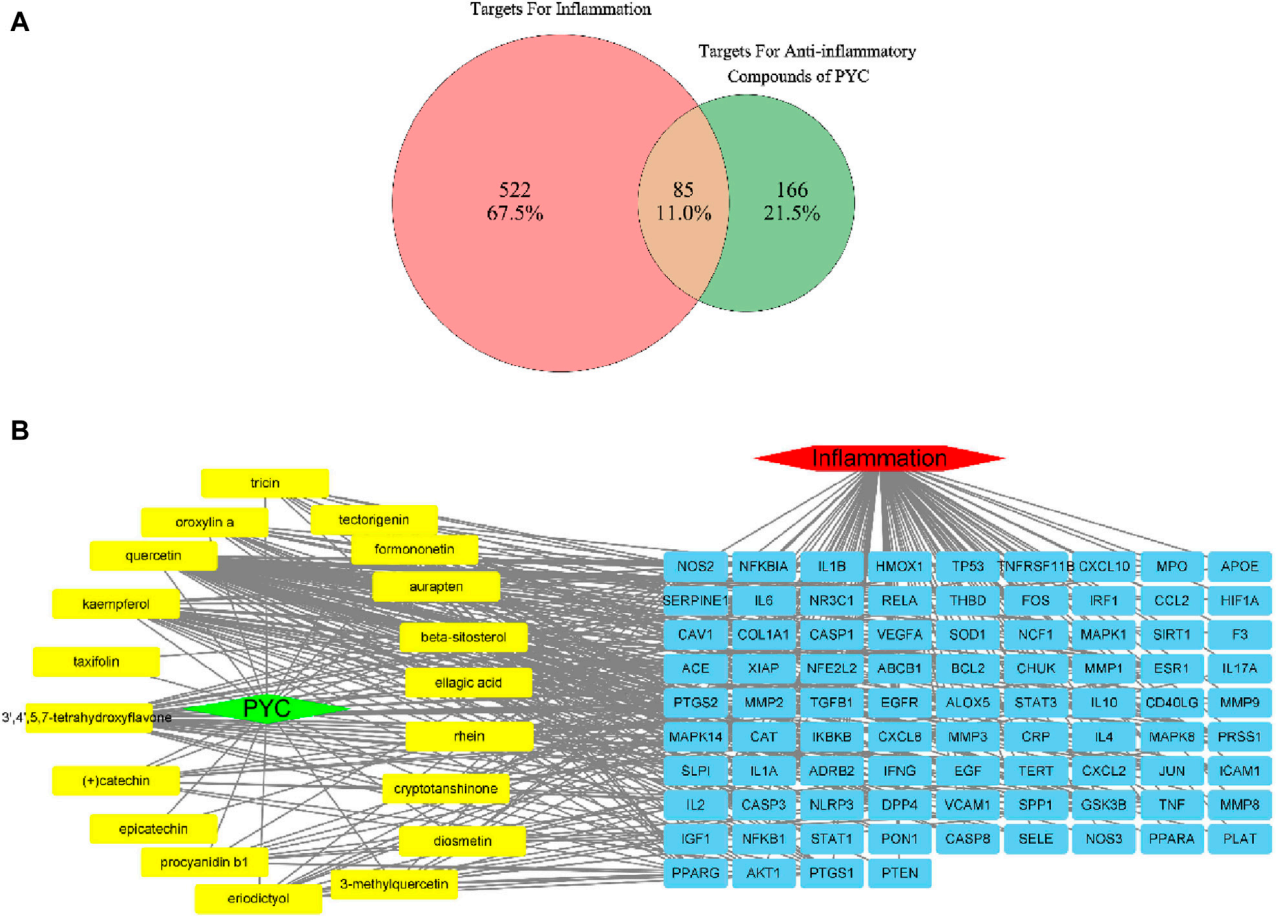
Targets of PYC’s anti-inflammatory compounds and inflammation. **(A)** Venn diagram of overlapping target genes between PYC’s anti-inflammatory compounds and inflammation. **(B)** PYC-compound-target-inflammation network. The yellow round rectangle nodes indicate the PYC’s anti-inflammatory compounds, the red hexagon node depicts inflammation, the green diamond node displays PYC, and the blue round rectangle nodes represent predicted PYC targets for treating inflammation.

HERB database identified 630 targets associated with the 19 anti-inflammatory compounds (Supplementary Table SA2). After duplicate removal, 251 unique targets remained for further analysis. From the GeneCards database, 607 inflammation-related targets were identified among 13,622 entries with a relevance score >5.0 (Supplementary Table SA3). Venn analysis identified 85 genes associated with PYC’s anti-inflammatory compounds and inflammation (Figure 4A), highlighting potential treatment targets. A network of 375 edges and 106 nodes was constructed to depict PYC-target-inflammation relationships (Figure 4B).

### 3.3 Analysis of the PPI network

**FIGURE 5:**
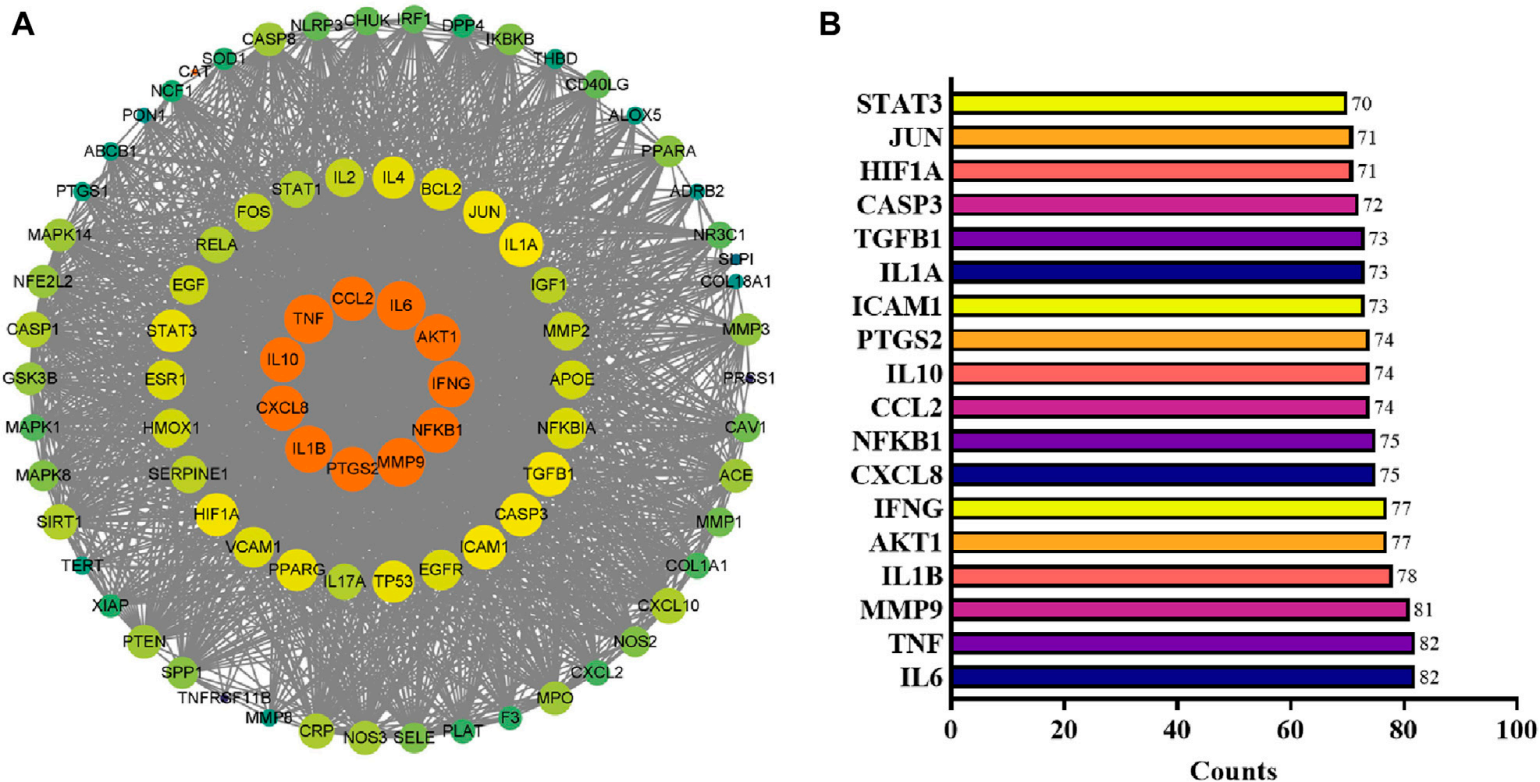
PPI network analysis. **(A)** PPI network reconstructed by Cytoscape. The node color and size are proportional to the target degree in the PPI network. **(B)** The degree values (≥70) of targets in the PPI network.

The PPI data for the specified targets were retrieved from the STRING database and imported into Cytoscape 3.9.1 to construct a function-related PPI network. Figure 5A shows the PPI network, which consists of 2,193 edges and 85 nodes. The average node degree was 51.6, and the edge referred to the interaction of nodes. After assessing each node’s degree value (Supplementary Table SA4), the gene with a degree value ≥54 (median) was identified as the key gene target, and a total of 43 genes were screened out. The top 18 targets with ≥70-degree value included IL6, TNF, MMP9, IL1B, AKT1, IFNG, CXCL8, NFKB1, CCL2, IL10, PTGS2, ICAM1, IL1A, TGFB1, CASP3, HIF1A, JUN, STAT3 (Figure 5B; the horizontal coordinate is the degree value of each target point).

### 3.4 Enrichment analysis

**FIGURE 6:**
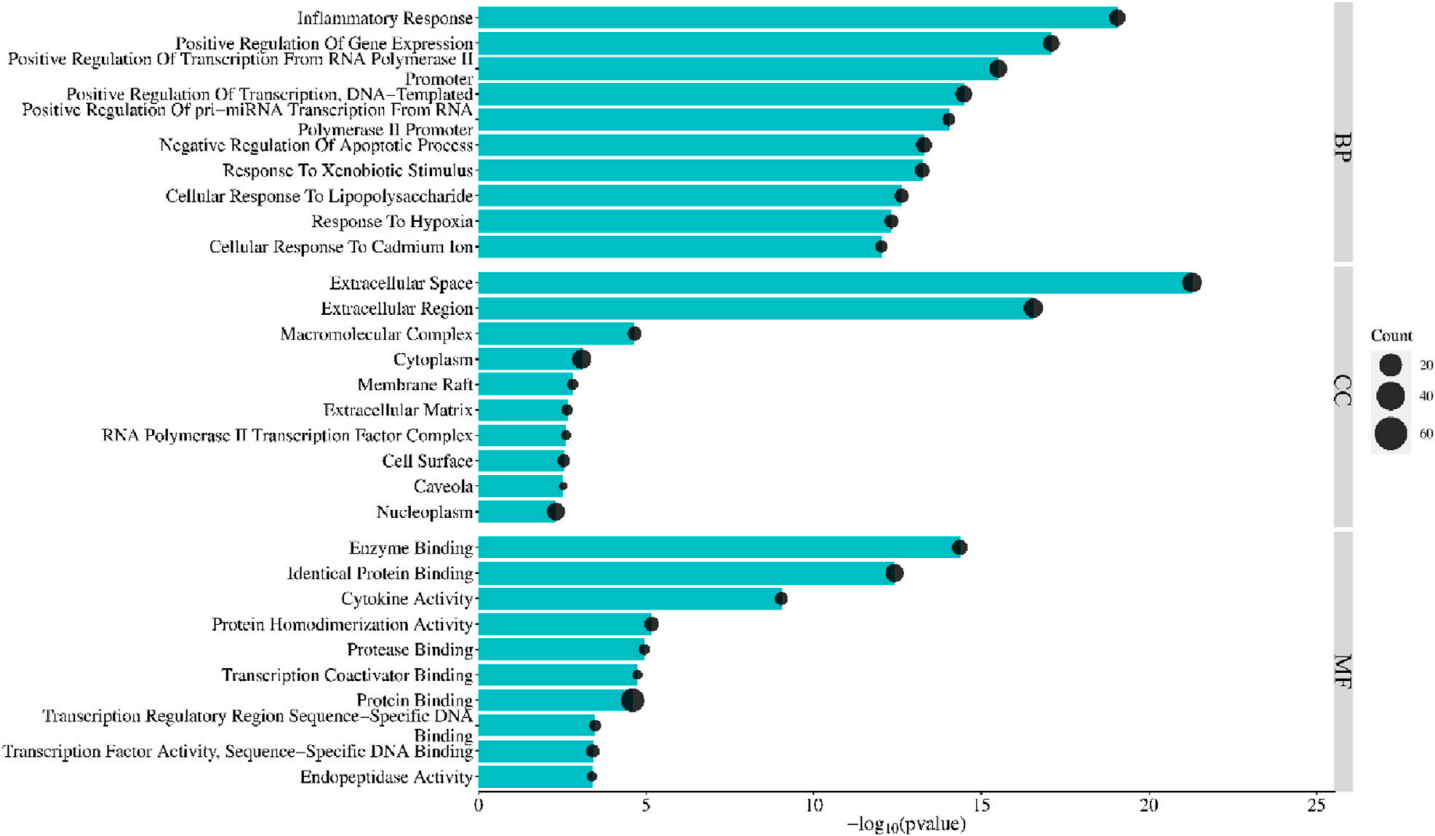
Top 10 GO terms of PYC candidate targets against inflammation.

Enrichment analysis using the DAVID database identified 85 PYC targets involved in treating inflammation. GO enrichment comprises cellular component (CC), biological process (BP), and molecular function (MF). Bonferroni-corrected *p*-*values* < 0.05 revealed 114 BP, 14 CC, and 22 MF (Supplementary Table SA5). Each category’s top 10 GO entries were selected according to the FDR <0.05 (Figure 6). The BPs enrichment items included positive transcription regulation, inflammatory response, positive regulation of transcription from RNA polymerase II promoter, positive gene expression regulation, negative apoptosis regulation*,* positive DNA-template modulation of pri-miRNA transcription from RNA polymerase II promoter, *etc.* The CCs enrichment items comprised extracellular region, macromolecular complex, cytoplasm, extracellular space, membrane raft, *etc*. The MFs enrichment items contained enzyme binding, cytokine activity, identical protein binding, protein homodimerization activity, protease binding, *etc.*

**FIGURE 7:**
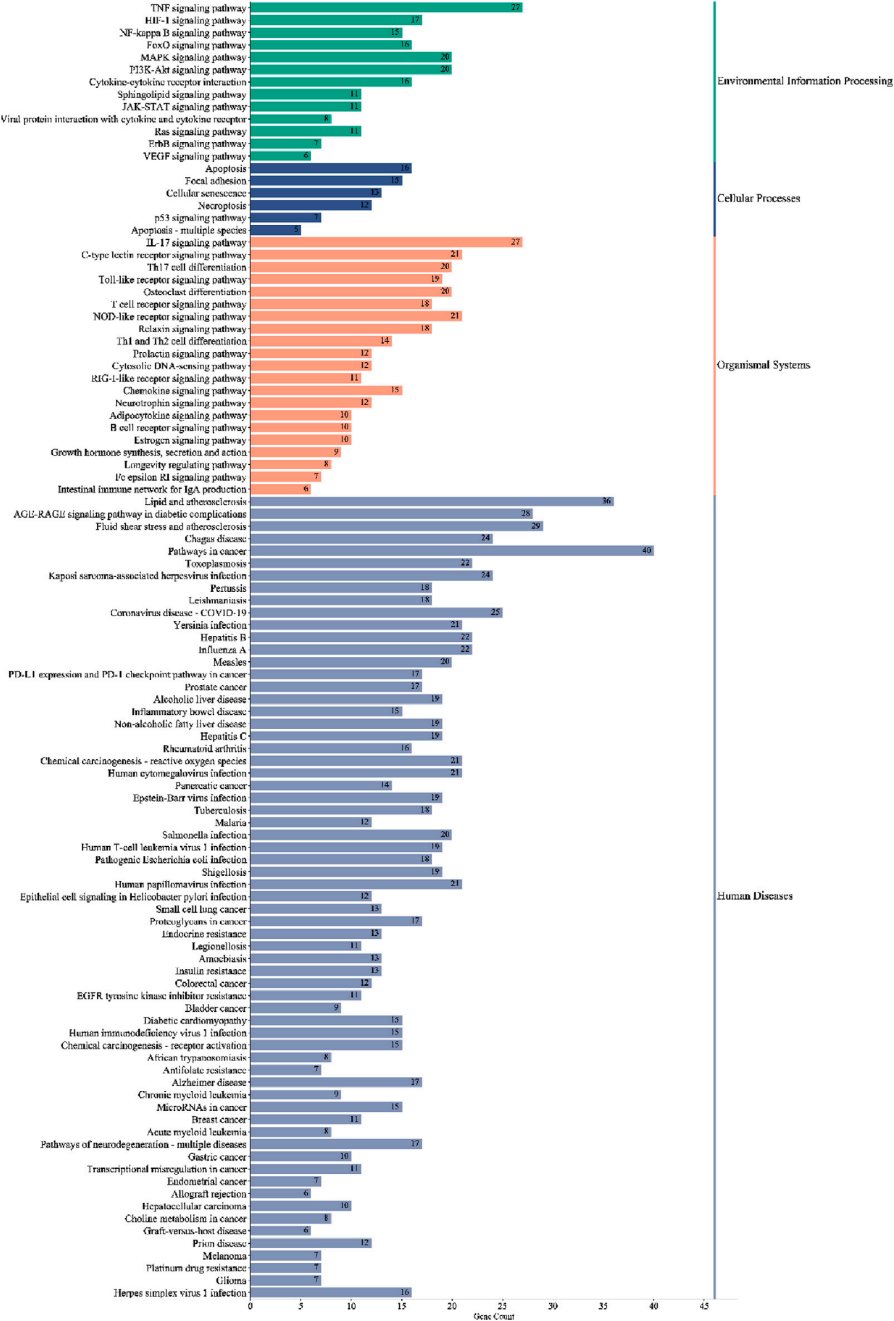
Classified enriched KEGG pathways of PYC candidate targets against inflammation. The pathways were classified into four major groups: human diseases, organismal systems, cellular processes, and environmental information processing.

**FIGURE 8:**
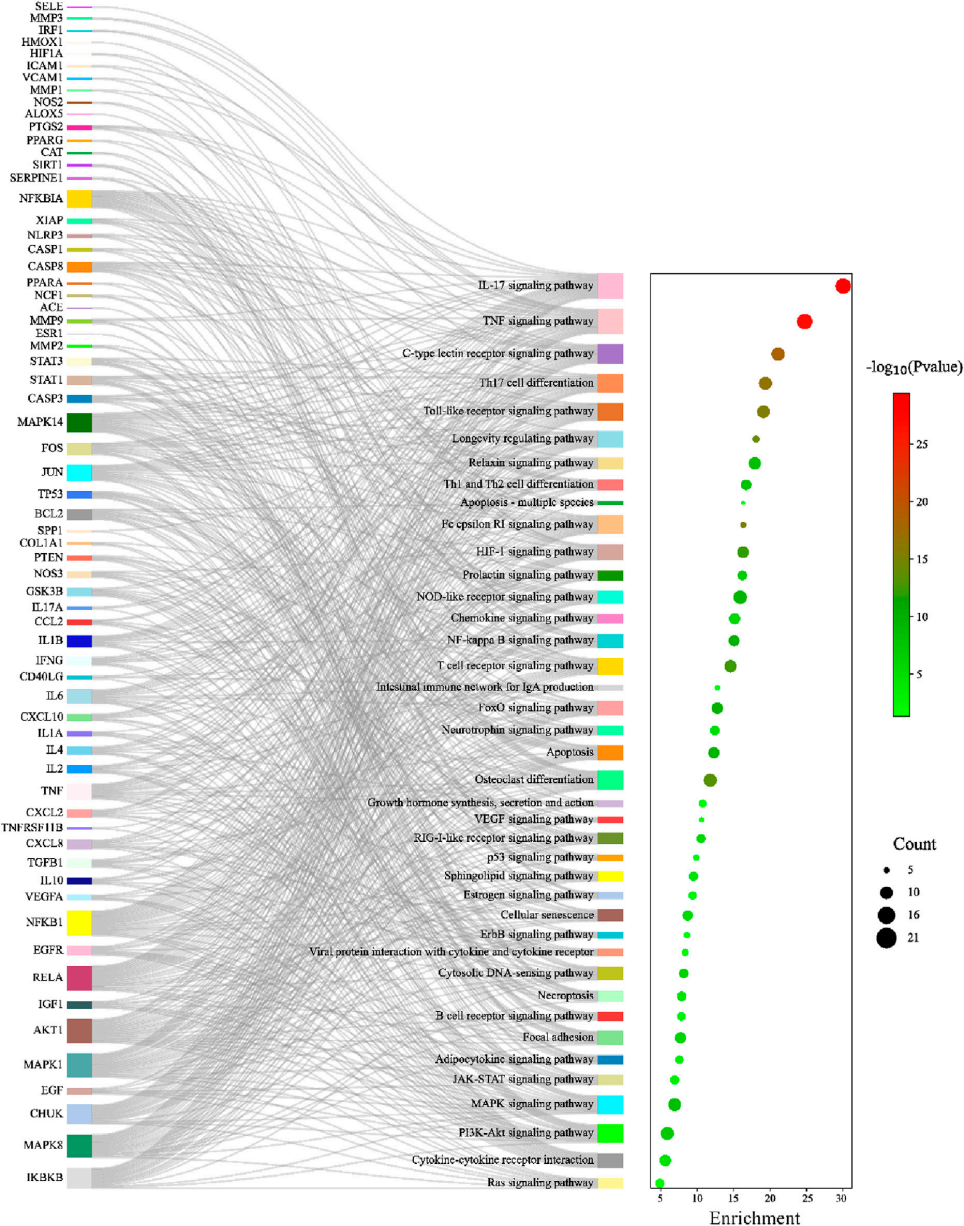
Pathway enrichment analysis of candidate targets of PYC against Inflammation.

KEGG pathway enrichment analysis identified 105 significant pathways with Bonferroni-corrected *p-value* < 0.05 (Supplementary Table SA6). These pathways were further classified into four major groups: six pathways of cellular processes, 21 of organismal systems, 13 of environmental information processing, and 65 were related to human disease (Figure 7). The pathways from the environmental information processing, organismal systems, and cellular processes groups were selected for the Sankey and dot plot chart: the IL17, TNF, C-type lectin receptor, Toll-like receptor, and NF-kappa B were the main signaling pathways associated with inflammation (Figure 8).

**FIGURE 9:**
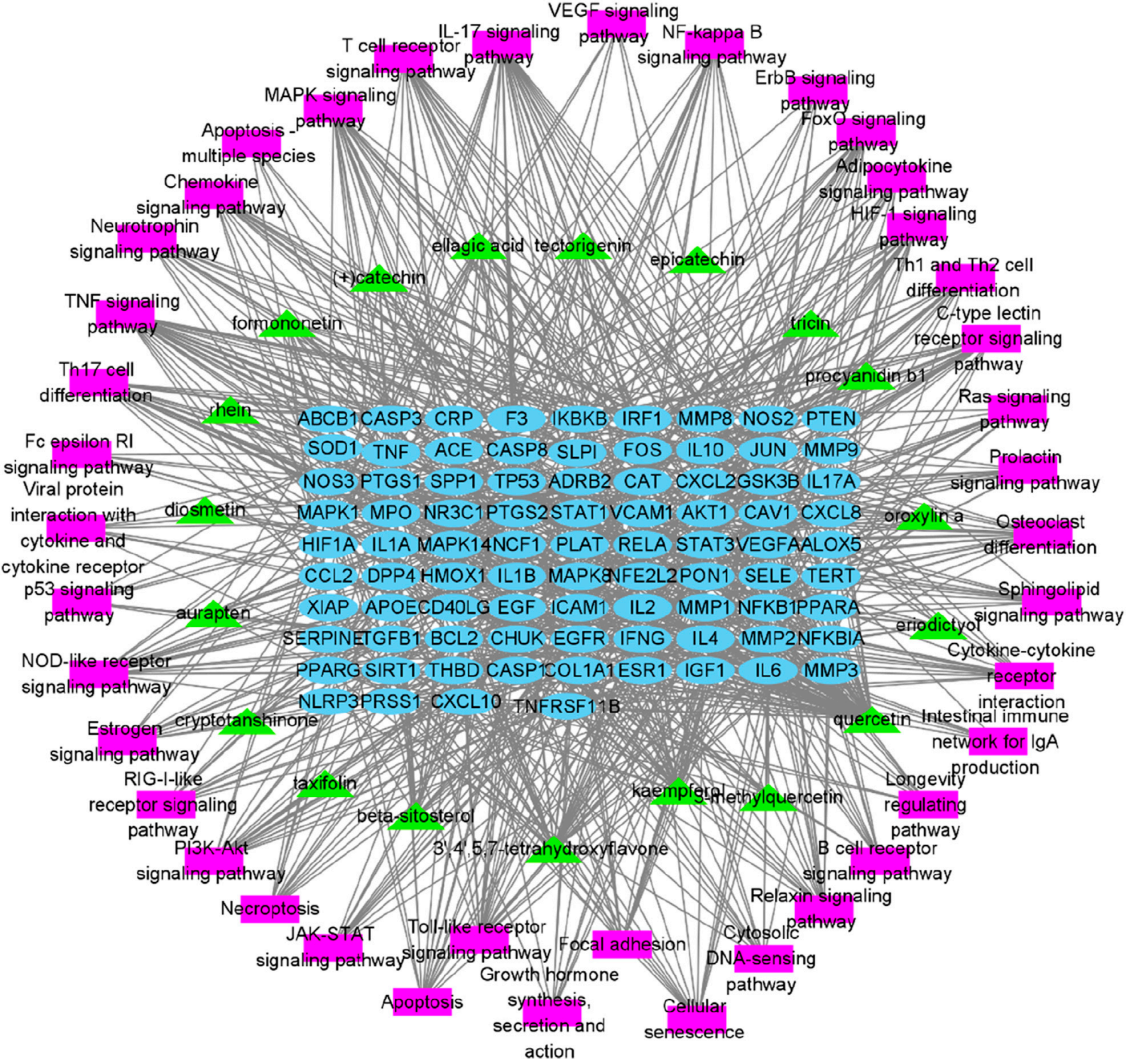
Network of compound-target-pathway. Green triangles, blue circles, and purple boxes represent compounds, targets, and pathways, respectively.

To further elucidate and understand the PYC pharmacological mechanism against inflammation, a network of 19 anti-inflammatory compounds, 85 anti-inflammatory targets, and 40 signaling pathways was constructed (Figure 9). This network indicated that the PYC’s components could regulate various targets, which regulates different signaling pathways.

### 3.5 The influence of PYC on cell viability

**FIGURE 10:**
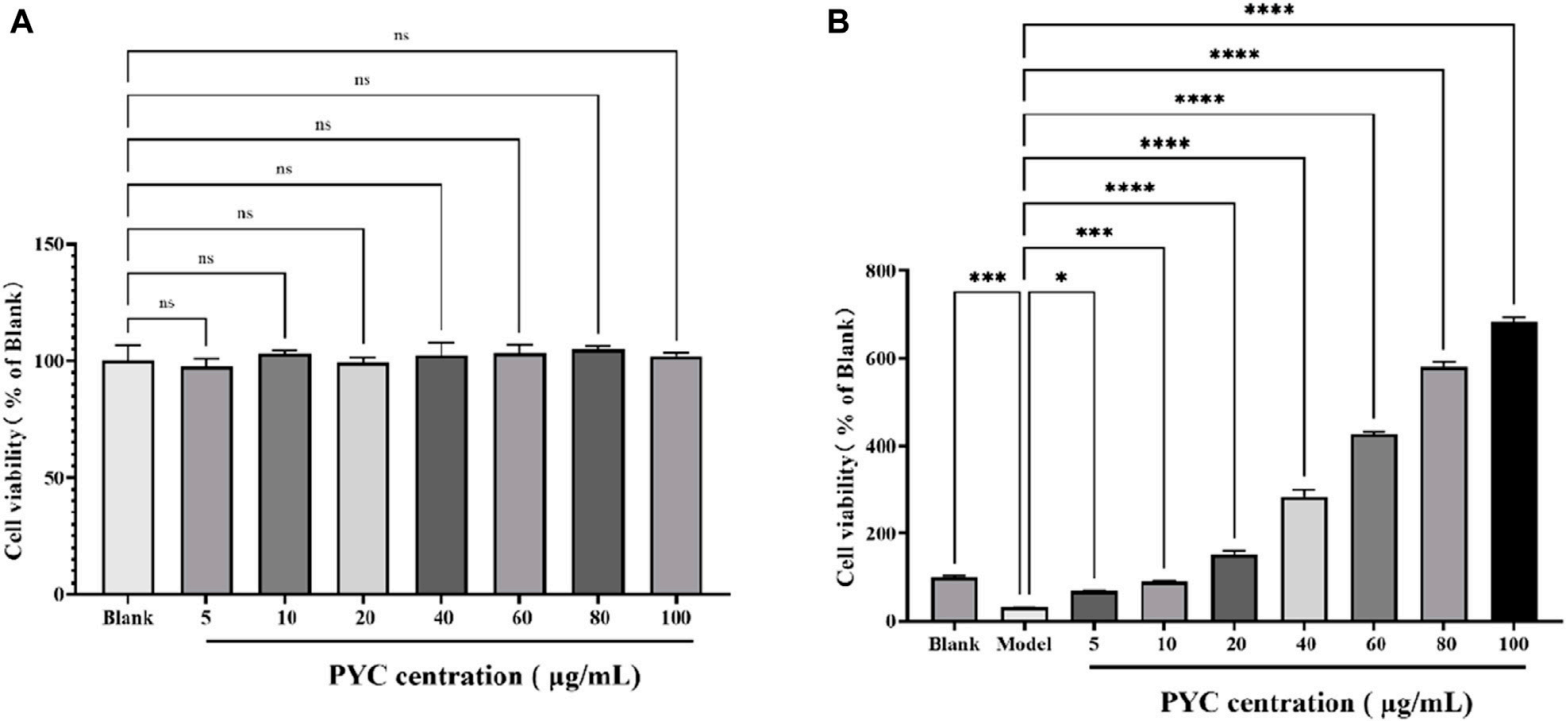
The influence of PYC on viability of BV2 cell **(A)** and the LPS-induced BV2 cell **(B)**.

The cell viability of BV2 cells is depicted in Figure 10. When treating BV2 cells with different concentrations of PYC, compared to the blank, there was no significant difference in cell viability, with the highest viability occurring at 80 μg/mL. Compared to the blank group (100% viability), the viability of the model cells significantly decreased to 30.96% (*p* < 0.001) but improved following PYC treatment, as shown in the PYC group (Figure 10B).

### 3.6 The PYC’s anti-inflammatory impact on LPS-induced BV2 cells

**FIGURE 11:**
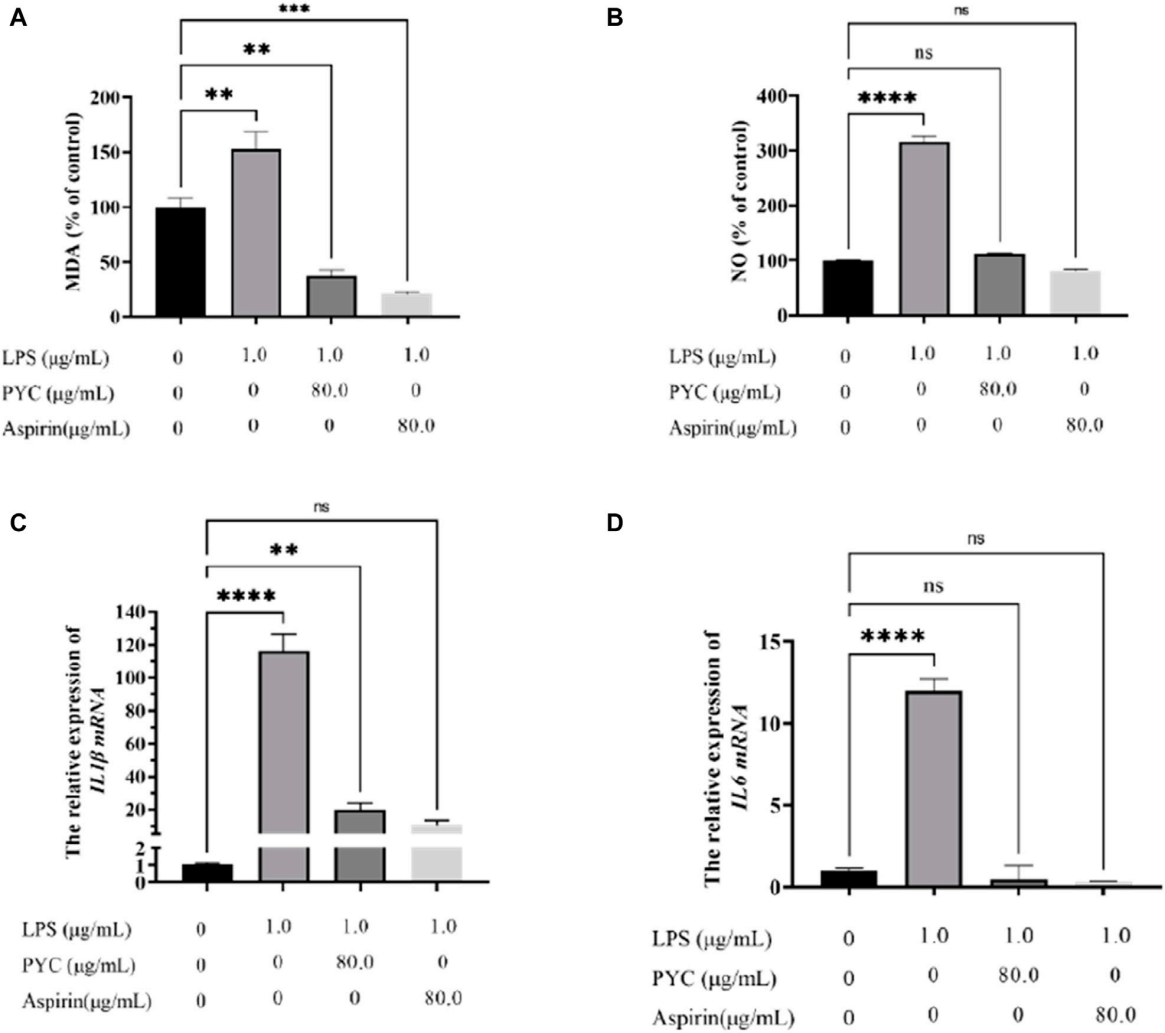
The effect of PYC on the levels of **(A)** Malondialdehyde (MDA), **(B)** NO, **(C)** *IL1β* mRNA, and **(D)** *IL6* mRNA.

MDA levels were significantly higher in the model cells than the controls but were markedly reduced after PYC and aspirin treatment (Figure 11A). Estimation of NO content and cytokines (IL1β and IL6) mRNA levels revealed significantly higher values in the model group compared to the control group. Furthermore, these levels were substantially decreased in the treatment group than in the model group (Figures 11B–D).

## 4 Discussion

Inflammation is a physiological response of the immune system to irritation, injury, or infection by pathogens, autoimmune processes, or radiation exposure. Controlled inflammation is beneficial as it helps remove irritants, damaged cells, and pathogens (Nakkala et al., 2021). However, dysregulated uncontrolled inflammation can cause several chronic diseases, even death (Ma and Willey, 2022). Glucocorticoids, broad-spectrum anti-inflammatory agents, are used to treat inflammatory diseases but are associated with several adverse effects, including high blood pressure, hyperglycemia, and adrenal insufficiency (Sun et al., 2022). Aspirin is a nonsteroidal anti-inflammatory drug that is known to act by blocking cyclooxygenase activity (Nyambuya et al., 2023), but a variety of side effects can be associated with long-term aspirin use, which include varying degrees of damage to the gastrointestinal tract, liver, kidney, and cardiovascular system (Shi et al., 2023). Thus, finding agents with substantial anti-inflammatory effects and few side effects is important. As an herb, pine bark extract is traditionally used as an anti-inflammatory in Europe with few side effects (D'Andrea, 2010).

Like macrophages, microglia are vital immune system components, stimulating proinflammatory cytokines involved in inflammatory responses (Xiong et al., 2016; Zhang et al., 2021). In order to better demonstrate the anti-inflammatory activity of PYC, based on our previous cytotoxicity experiments, we chose the concentration of PYC as 80 μg/mL, which is above any concentration that can be expected *in vivo* (except for concentrations in the intestine). This study identified the inhibitory effects of PYC on the levels of NO and proinflammatory IL6 and IL1β cytokines in LPS-stimulated microglia (Figure 11). Network pharmacology analysis identified 19 anti-inflammatory compounds in PYC. PYC is recognized as one of the most powerful natural antioxidants, which is rich in flavonoids, phenolic acids and proanthocyanidins. The aqueous extract of PYC contains (+)-catechin, (−)-epicatechin, (+)-taxifolin, the benzoic acids protocatechuic acid, vanillic acid, gallic acid, the cinnamic acids, ferulic acid, caffeic acid, p-coumaric acid and higher molecular weight procyanidins predominate (Rohdewald, 2002; Bayer and Högger, 2024). Except for (+)-catechin, (−)-epicatechin, (+)-taxifolin and procyanidin b1, the other fifteen molecules we listed here are novel. While the compounds first reported here such as ellagic acid and quercetin which were a typical polyphenolic compound and flavonoid found in various plants (Vattem and Shetty, 2005; Singh et al., 2021). PYC is a bark extract of the French maritime pine (*Pinus pinaster*), hence the detection of these compounds is reasonable. The compounds listed here are related to anti-inflammatory ingredients after network pharmacology integrating and other detected chemicals of PYC extraction supernatant are not presented. Among them, 12 compounds (3′,4′,5,7-tetrahydroxyflavone, 3-methylquercetin, eriodictyol, procyanidin b1, epicatechin, (+) catechin, diosmetin, taxifolin, kaempferol, quercetin, oroxylin-a, and tricin) were flavonoids, tectorigenin, and formononetin were iso-flavonoids. Flavonoids comprise two benzene rings linked with a heterocyclic pyran or pyrone ring. These naturally occurring polyphenolic compounds have diverse biological activities. Compounds such as eriodictyol, procyanidin B1, epicatechin, (+)-catechin, taxifolin, kaempferol, quercetin, and tricin have demonstrated anti-inflammatory properties (Morikawa et al., 2006; Moscatelli et al., 2006; Hämäläinen et al., 2007). 3′,4′,5,7-tetrahydroxyflavone, known as luteolin, suppresses LPS-mediated inflammatory responses by modulating NF-κB/AP-1/PI3K-Akt signaling cascades (Park and song, 2013). Oroxylin-a suppresses LPS-mediated gene expression of COX-2 and iNOS by inhibiting NF-κB activation (Chen et al., 2000). Tectorigenin and formononetin, as isoflavonoids, can also display the inhibitory effects of NO release in LPS-activated mouse peritoneal macrophages (Matsuda et al., 2003). Ellagic acid, a phenolic acid, significantly inhibited NO in RAW 264.7 cells (Yang et al., 2011). Furthermore, Rhein can effectively suppress NO release and substantially reduce the expression of iNOS and COX-2 proteins after 18 h of LPS treatment (Choi et al., 2013). In RAW264.7 cells, cryptotanshinone significantly suppressed LPS-mediated NO generation and cytokines release like IL6 and TNFα (Gao et al., 2018). Auraptene and beta-sitosterol also showed anti-inflammatory effects (Morikawa et al., 2006; Nguyen et al., 2016). These studies further confirm the potential of the 19 anti-inflammatory compounds identified in PYC.

For the 19 identified anti-inflammatory compounds, 85 target genes were identified. PPI analysis and network pharmacology revealed core targets including IL6, TNF, MMP9, IL1B, AKT1, IFNG, CXCL8, NFKB1, CCL2, IL10, and PTGS2. Of these, IL6, TNF, IL1B, IFNG, CXCL8, and IL10 are the proinflammatory cytokines usually employed for assessing the anti-inflammatory effects of drugs (Xia et al., 2023). Studies have shown that levels of IL1β, IL6, and TNF are significantly associated with the intensity of systemic inflammation (Del Valle et al., 2020). MMP9, usually expressed in inflammatory cells, is a known activator of inflammatory cytokines like TNF and IL1β (Cheeran and Munuswamy-Ramanujam, 2020). AKT1, a significant subtype of serine/threonine kinase (AKT), regulates macrophage innate immunity by producing mitochondrial H_2_O_2,_ and its levels are crucial for stimulating inflammatory reactions (Tang et al., 2017). NFKB1 is a pleiotropic transcription factor in almost all cell types and has been indicated as a crucial inflammatory regulator (Tu et al., 2020). CCL2 exerts its effect as an inflammatory chemokine, essential for recruiting leukocytes and memory T cells during inflammation (Ranjbar et al., 2022). PTGS2 expression is promoted during inflammation and regulates inflammatory responses by generating prostaglandins (Li et al., 2022). Usually, PYC can reduce inflammation by acting as a blocker of inflammatory transcription factors NF-kB and lowering the levels of MMP-9 (Grimm et al., 2006), which is consistent with our analysis results for the PYC’s target. When pycnogenol is administered, are lowered, displaying the inhibitory effect of pycnogenol on NF-kB All identified core targets were closely associated with the inflammation.

The KEGG and GO enrichment tests predicted the potential functions of the 85 targets and signaling pathways associated with the anti-inflammatory response of PYC. The GO analysis revealed that the BP of the targets was mainly involved in the inflammatory response, transcription regulation, and cell response; the CC affected by the targets included extracellular structures, and the MF of the targets was binding and cytokine activity (Figure 5A). Furthermore, it was indicated that PYC’s anti-inflammatory compounds are involved in various biological processes, cellular components, and molecular functions as defined in the GO categories. KEGG enrich analysis suggested that the anti-inflammatory compounds in PYC might exert their anti-inflammatory effect through the IL17 and TNF signal pathways. Interestingly, among the 11 core targets (IL6, TNF, MMP9, IL1B, AKT1, IFNG, CXCL8, NFKB1, CCL2, IL10 and PTGS2) analyzed by PPI, 10 genes except IL10 participated in IL17 signaling pathway. This pathway is initiated after the interaction of cytokine (IL17A/F, IL17F/F, or IL17A/A) with their receptors IL17RC and IL17RA, which activates TRAF proteins, which in turn stimulate C/EBPβ, NF-κB, C/EBPδ, and MAPK pathways, aggravating inflammation (Amatya et al., 2017). Contrasting with the IL17 signaling pathway, the TNF signaling pathway, a traditional proinflammatory pathway, involves six core targets: IL6, TNF, MMP9, IL1B, AKT1, and CCL2. Furthermore, it was found that PYC treatment reduced the expression of IL6 and IL1B, thereby alleviating inflammation.

Traditional anti-inflammatory agents usually act on a single target, which can lead to drug resistance during long-term use. For example, dexamethasone binds to the cytoplasmic GC receptor to form a heterodimer that translocated from the cytosol to the nucleus, where it transactivates or transrepresses target genes, leading to cell-cycle arrest and apoptosis (Zhang et al., 2023); aspirin is known to block cyclooxygenase activity (Nyambuya et al., 2023). Therefore, treatment of the chronic inflammation, dexamethasone and aspirin showed no effect (Thuo et al., 2022). Different from traditional anti-inflammatory agents, PYC could affect inflammation via multi-components, -targets and -mechnism, which avoid drug resistance and is suitable for long-term use. Moreover, The results shown in this *in vitro* study demonstrate that PYC was observed to be non-toxic shown by the cytotoxicity analyses in BV2 cells at the conctration below 100 μg/mL. Other studies also shown that no acute toxicity was identified with intraplantar injection of P. brutia bark extract into rat at a dose of 2000 mg/kg body wt (Ince et al., 2009). Therefore, PYC can be utilized as an anti-inflammatory agent.

Nevertheless, the study still presents certain limitations. Our research primarily centers on investigating the anti-inflammatory properties of the compounds found in PYC. We have solely examined the direct anti-inflammatory effects of these compounds in PYC, neglecting to explore the potential anti-inflammatory effects of their metabolites post-assimilation in the body. Consequently, a comprehensive understanding of the overall anti-inflammatory potential of PYC remains elusive, and and further *in vivo* and *in vitro* research need to be conducted.

## 5 Conclusion

This study systematically analyzed 768 chemical components in PYC using UPLC-MS/MS. After that, 19 anti-inflammatory compounds targeting 85 proteins directly involved in inflammation were identified using the InflamNat and TCMSP databases. Furthermore, PPI analysis indicated the core targets, including IL6, TNF, MMP9, IL1B, AKT1, IFNG, CXCL8, NFKB1, CCL2, IL10, and PTGS2. GO analysis indicated that these targets were involved in the BP, such as inflammatory response, transcription regulation, and cell response; they affected the CC, mainly the extracellular structures, and their MF included cytokine activity and binding. KEGG pathway analysis suggested that the anti-inflammatory compounds in PYC may exert their effects through the IL17 and TNF signaling pathways. Furthermore, it was found that PYC treatment reduced the expression of IL6 and IL1B, thereby alleviating inflammation. However, further research is required to elucidate the underlying mechanisms fully.

## Data Availability

The original contributions presented in the study are included in the article/Supplementary Material, further inquiries can be directed to the corresponding author.

## Abbreviations

PYC, Pycnogenol; UPLC-MS/MS, Ultra-high performance liquid chromatography-tandem mass spectrometry; InflamNat, Inflammatory Natural Products Database and Predictor; TCMSP, Traditional Chinese Medicine Systems Pharmacology Database and Analysis Platform; PPI, Protein-Protein Interaction; KEGG, Kyoto Encyclopedia of Genes and Genomes; LPS, Lipopolysaccharide; CCK8, Cell Counting Kit 8; MDA, Malondialdehyde; NO, Nitric Oxide; DL, Drug-Likeness; OB, Oral Bioavailability; RBN, Rotatable Bond Number; HERB, High-throughput Experiment- and Reference-guided database of TCM; CTD, Comparative Toxicogenomics Database; GO, Gene Ontology; DAVID, Database for Annotation, Visualization, and Integrated Discovery; FDR, False Discovery Rate; OD, optical density; TBA, Thiobarbituric Acid; SEM, Standard Error Of The Mean.
